# Cumulative Radiation Dose from Medical Imaging in Children with Congenital Heart Disease: A Systematic Review

**DOI:** 10.3390/children10040645

**Published:** 2023-03-30

**Authors:** Emer Shelly, Michael G. Waldron, Erica Field, Niamh Moore, Rena Young, Andy Scally, Andrew England, Michael Maher, Mark F. McEntee

**Affiliations:** 1 Discipline of Medical Imaging & Radiation Therapy, University College Cork, T12AK54 Cork, Ireland; 2 Department of Radiology, Cork University Hospital, T12 DC4A Cork, Ireland; 3 Department of Medicine, University College Cork, T12 AK54 Cork, Ireland

**Keywords:** cumulative, radiation dose, medical imaging, congenital heart disease

## Abstract

Children with congenital heart disease are exposed to repeated medical imaging throughout their lifetime. Although the imaging contributes to their care and treatment, exposure to ionising radiation is known to increase one’s lifetime attributable risk of malignancy. A systematic search of multiple databases was performed. Inclusion and exclusion criteria were applied to all relevant papers and seven were deemed acceptable for quality assessment and risk of bias assessment. The cumulative effective dose (CED) varied widely across the patient cohorts, ranging from 0.96 mSv to 53.5 mSv. However, it was evident across many of the included studies that a significant number of patients were exposed to a CED >20 mSv, the current annual occupational exposure limit. Many factors affected the dose which patients received, including age and clinical demographics. The imaging modality which contributed the most radiation dose to patients was cardiology interventional procedures. Paediatric patients with congenital heart disease are at an increased risk of receiving an elevated cumulative radiation dose across their lifetime. Further research should focus on identifying risk factors for receiving higher radiation doses, keeping track of doses, and dose optimisation where possible.

## 1. Introduction

Congenital heart disease is a defect in the structure of the heart or the great vessels, which is present at birth and occurs in approximately 1% of births per year in the United States [1]. Many of the diagnostic and interventional tools used to investigate and treat congenital heart disease involve the use of medical ionising radiation. The main concern with these procedures is the potential increase in the lifetime attributable risk of radiation-induced malignancy [2]. Patients with congenital heart disease are often exposed to relatively high doses of ionising radiation from medical sources from a very young age, often even from birth [3]. Malignancy due to ionising radiation poses a particular risk to children due to their rapidly dividing cells and longer life span in which the radiation can potentially cause damage [4]. Awareness of radiation safety and its possible long-term effects is suboptimal among many doctors, including paediatricians [5,6].

Lifetime cumulative radiation dose and the associated risk of developing cancer is a poorly understood subject. Much of our current understanding of the effects of radiation is based on atomic bomb survivors [2] or studies based on large single doses of radiation of >100 mSv [7]. Recent evidence would suggest that a high cumulative dose does carry an increased risk of developing cancer [8]. However, it remains unclear if this risk is the same as receiving the equivalent amount of radiation in one dose. Notably, according to the linear no-threshold (LNT) hypothesis, any dose, irrelevant of size, carries an inherent risk [9,10]. Therefore, the cumulative exposure burden to children with congenital heart disease calls for better understanding and awareness so that those involved in their care can make more informed decisions and, where possible, use a dose as low as reasonably acceptable.

The importance of understanding the risks associated with repeated exposure to ionising radiation, particularly in childhood, is emphasised by epidemiological studies. Researchers analysed the data of 11 million Australians to assess the malignancy risk in children and adolescents by analysing cancer incidence rates in those who had a CT more than one year before diagnosis compared to a group unexposed to CT imaging prior to malignancy diagnosis. They highlighted an elevated risk with an absolute excess incidence of 9.38 per 100,000 person-years at risk [11]. It must, however, be recognised that many of these types of studies have limitations, such as the dose estimation methods (which may over/underestimate risks), and the eventual lifetime risk of cancer is difficult to estimate due to the time limits of the studies (which may minimise risks).

The Alliance for Radiation in Paediatric Imaging has recognised the importance of monitoring low-dose ionising radiation in the “Image Gently” campaign to improve safe and effective imaging care for children worldwide. Image Gently advocates methods to reduce unnecessary ionising radiation by sharing best practices of imaging protocols for children and using alternative imaging that avoids ionising radiation. They have also created informational and advocacy information for each ionising radiation modality [12]. More recently, in 2017, a document from the Image Gently Alliance highlighted the relatively high cumulative lifetime burden of ionising radiation in children with complex congenital and acquired heart disease (CAHD) from the multiple imaging studies and procedures over their lifetime [3]. In this document, they emphasised the need to achieve high-quality studies with the lowest achievable radiation dose and to standardise dose metrics across imaging modalities to encourage comparative effectiveness studies across the spectrum of CAHD in children.

Many efforts have been made so far to reduce the radiation dose to CHD patients [13,14,15], including an interesting study by Patel et al. [16], which implemented several system-based interventions in the congenital cardiac catheterisation lab to reduce radiation exposure to paediatric CHD patients, including the utilisation of lower fluoroscopy and digital angiography doses, increasing staff and physician awareness, focusing on tighter collimation, and changing the default fluoroscopy and DA doses to lower settings [17]. Studies like these demonstrate the potential change that is possible on a broader scale to reduce radiation dose, ultimately lowering the cumulative radiation burden that CHD patients are exposed to.

The concepts of justification and the ‘As Low as Reasonably Achievable’ (ALARA) principle are imperative in medical imaging, especially regarding patients repeatedly exposed to radiation. A study assessing the rate of patients receiving high cumulative doses over 100 mSv highlighted an incidence of 0.21% over five years across 35 countries [18]. An up-to-date systematic review must be undertaken to establish the CED that this cohort of patients is receiving in a wide variety of settings, in addition to the modalities utilised and conditions that put these patients at a higher risk of receiving a higher cumulative radiation dose. This study aims to systematically review published data regarding the cumulative exposure from medical sources of ionising radiation in paediatric patients with CHD. We aim to provide the medical imaging community with a better understanding of dose estimation, cumulative dose, and risk factors for CHD patients who may be exposed to higher/repeated radiation. This will allow future practice to be improved and further work to be done surrounding the monitoring and reducing the cumulative dose of these patients.

## 2. Materials and Methods

The systematic review was performed in accordance with the pre-specified protocol, which was registered on PROSPERO, the prospective international register of systematic reviews.

### 2.1. Search Strategy

A comprehensive search of the current literature was performed in accordance with the Preferred Reporting Items for Systematic Reviews and Meta-Analyses (PRISMA) guidelines [19]. PubMed, Science Direct, Scopus, and Embase were searched. The ‘year published’ filter was applied for each database to only capture studies published between 1 January 2010 and 1 March 2023. Several pre-determined keywords were pooled for the systematic search and subsequent MeSH terms were generated. The MeSH terms used were “cumulative effective dose”, “cumulative dose”, “cumulative exposure”, “cumulative radiation dose”, “cumulative medical radiation exposure”, “CHD”, “congenital heart disease”, “pediatric”, “paediatric”, “child”, and “children”. The MeSH terms were then combined according to the specifications of each database using link words such as “AND”, “OR”, and “NOT”.

### 2.2. Data Synthesis

An initial screen of the literature was performed using the titles and abstracts of all articles that had been identified. Only articles that contained data from cumulative radiation dose in a follow-up period of one year minimum for children aged ≤ 18 years were included for a full-text review. Meta-analysis and review articles were excluded. For each study, reviewers applied inclusion/exclusion criteria as outlined below:

Clinical trials and observational studies were published between 2010 and 2020 in the English language.

(1) Only studies involving a minimum of 20 paediatric patients.
(2) Only studies involving the monitoring of cumulative radiation dose over at least one year.
(3) Only studies involving the monitoring of cumulative radiation dose from medical sources (both interventional and diagnostic) as opposed to other sources.
(4) Only studies where the patients had some form of congenital heart disease and/or had several cardiac interventional/diagnostic procedures carried out.
(5) Only studies where the outcome measures included the cumulative radiation dose of included patients.
(6) Only original cross-sectional studies, case-control studies, cohort studies, or randomised control trials.

Quality assessment was carried out using the STROBE quality assessment tool [20], and a risk of bias was applied to all included studies [21].

## 3. Results

### 3.1. Study Selection and Quality Assessment

**Figure 1:**
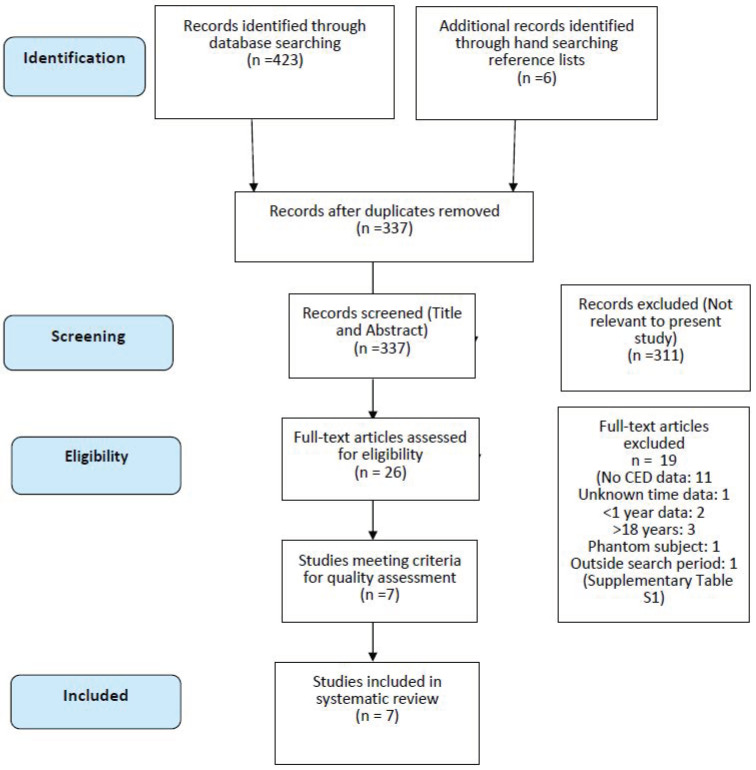
PRISMA flow diagram.

The systematic search revealed 296 papers from PubMed/Science Direct, 66 from SCOPUS, and 61 from EMBASE. Hand-searching reference lists also found six further studies from other papers. After removing the duplicates, 337 titles and/or abstracts were assessed for relevance, and this resulted in 26 papers which were relevant to this review question. The full text of these 20 articles was reviewed, and inclusion/exclusion criteria were applied. Two reviewers (ES and EF) carried out this step to reduce bias. Any discrepancies at this stage were discussed, and updates were made where necessary. Seven of these 20 papers were agreed upon to be eligible for inclusion in this systematic review (Figure 1) (Supplementary Table S1).

Qualitative analysis was applied to these seven papers using the STROBE quality assessment tool by two independent reviewers (ES and EF). According to STROBE, all seven studies reached an acceptable level of quality. Certain limitations were identified outside of STROBE that included potential unrecorded radiation doses for investigations performed outside the facility of the study. The sample size for each paper was spread across an extensive range. Some papers had a low sample size (McDonnel et al. sampled 31 patients), with some cohorts followed for a relatively short time (Ait-Ali et al. [22] followed patients for one year). These factors need to be taken into consideration when interpreting the results. The level of homogeneity across these studies also proved to be a challenge, with several papers presenting their outcomes differently. Also, we assessed papers performed across a range of modalities that these papers collected data, which allowed us to understand the overall picture of the cumulative radiation dose of CHD patients from all their medical imaging.

### 3.2. Data Extraction

**Figure 2:**
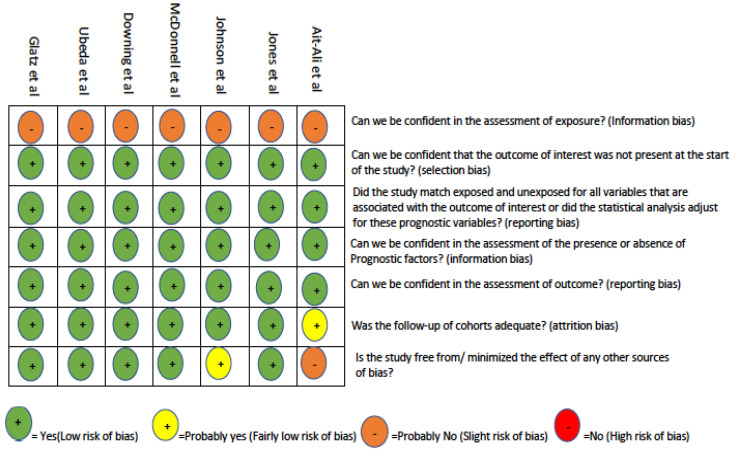
Risk of bias [22,23,24,25,26,27,28].

A risk of bias assessment (Figure 2) was performed using questions designed to review cohort studies developed by the Clarity Group at McMaster University [21]. The overall risk of bias across all seven studies was relatively low. Due to the nature of the assessment of exposure and the variation of estimation techniques across the studies, there was likely a degree of information bias across all seven studies. It is also important to note that the exposure assessment may represent some bias since the patients were only followed in a single centre except for Ait-Ali et al. [22]. Assessment of confidence in the outcome was high across all papers but may be slightly lower in Ait-Ali et al., since the lifetime radiological exposure was derived from patient records. This may cause an underestimation in the calculation of the cumulative radiation dose.

Table 1 summarises the cumulative radiation exposure of included studies, the study type, the number of patients, the x-ray procedures recorded, and the effective dose estimation method. The contribution of different imaging modalities to the cumulative recorded radiation dose is recorded (Supplementary Table S2).

## 4. Discussion

The seven included papers [22,23,24,25,26,27,28] utilised CED to measure ionising radiation exposure. CED is the total dose resulting from repeated exposures to ionising radiation from all radiologic studies [29]. Translating the relatively low levels of medical ionising radiation these patients receive into mutagenic effects is difficult due to sparse data, as most of our understanding of cancer risks stems from data on atomic bomb survivors [8]. Although the elevated risk associated with a single large radiation dose is known, the effects of recurrent smaller exposures are not well defined. However, the linear no-threshold model (whereby the risk of cancer increases linearly with the increase in radiation dose with no lower radiation dose limit) is widely accepted and provides the best description of the relationship between ionising radiation dose received and the risk of stochastic effects. Over 85% of infants born with modern or complex heart disease live to adulthood, with medical imaging involving ionising radiation playing a significant role in their diagnosis, treatment, and survival [30]. A large population-based study by Mathews et al. [11] suggested that each sievert of effective dose from CT scans can be attributed to 0.125 excess cancers in an average follow-up of 9.5 years. Therefore, it is more imperative than ever to assess and manage the doses being administered to these patients, following ALARA principles.

Unsurprisingly, diagnostic and interventional cardiac catheterisation represented the most significant percentage of cumulative effective dose across all seven included studies. In a previous study, the median exposure was reduced by 30% for all cases by employing institution-specific quality improvement intervention techniques [31]. Conversely, radiographs represented the lowest contribution to cumulative exposure across all studies where it was recorded. Although CT was only recorded in two papers, it represented a small percentage of examinations in both cases whilst contributing a large proportion of the overall radiation dose. CT is frequently used in diagnosing and treating CHD [32] and is a significant contributor to CED. Therefore, further studies should focus on reducing CED in CHD patients. Substantial efforts have been made to manage the radiation dose for paediatric cardiac CT via the Image Gently ‘Have-a-Heart’ campaign [33]. They found that understanding CT technical parameters and how to apply them to children of various sizes and heart rates is necessary to optimise image quality at the lowest possible radiation dose. A CT dose management program should be implemented to ensure regulatory compliance and to optimise ionising radiation use in patients exposed to long-term cumulative radiation due to their high risk of repeated and elevated exposure. Currently, the limit on effective dose for occupational exposure is 20 mSv, according to the ‘Radiological Protection Act 1991- Ionising Radiation Regulation S.I No. 30 2019’ [34]. It is, therefore, even more crucial that we consistently monitor and assess every single dose of ionising radiation and whether it can be reduced or avoided altogether.

There are many ways in which dose reduction can be achieved. These are generally classified as justification, optimisation, and dose limitation. iRefer guidelines [35] and the European Society of Radiology iGuide [36] tools can aid clinician decision-making for the justification of the use of medical imaging with suggestions for decisions including information related to radiation reduction and cost efficiency. Appropriate use of ionising radiation is essential in minimising the cumulative radiation dose to CHD paediatric patients. Optimisation strategies across different imaging modalities are possible. For example, in cardiac CT, the lowest practical radiation dose which can achieve acceptable image quality should be used. The use of ECG-gated tube current modulation for functional imaging, can greatly reduce dose [3]. In nuclear cardiology, using advanced hardware (e.g., PET, CZT) or software technology to reduce administered activity can also aid in dose optimisation [3]. The introduction of iterative algorithms in CT reconstruction offers promise of further reduction in the radiation dose to CHD patients. The second way of reducing dose can be by using alternative modalities which do not use ionising radiation, such as ultrasound or magnetic resonance imaging. Echocardiography is the first-line imaging technique used for CHD, as it is capable of providing excellent depiction of intracardiac and valvular anatomy, cardiac function, and hemodynamic [29]. Dose reduction can also be implemented with the help of diagnostic reference levels (DRLs). There is currently limited data in Ireland for DRLs for paediatric catheterisation. However, Eurosafe Imaging has provided a document on European DRLs for paediatric imaging that can assist dose optimisation [37].

A limitation of this review is that all studies assessing CED in CHD were included (both diagnostic and therapeutic). Some studies derived the cumulative effective dose from solely cardiac procedures as opposed to from all imaging modalities [27,28], which may represent an underestimation. This increased the heterogenicity between the studies, as such a meta-analysis could not be performed and increased difficulties in comparing the studies. A multi-centre study is required to gain a more accurate estimation of CED, with a separate investigation of radiation from diagnostic and interventional procedures (and overall CED), which should be aided by a state-wide dose estimation programme. A strength of this study was that the risk of bias within the review was kept as low as possible with two reviewers carrying out the search strategy, quality assessment, data extraction, and risk of bias assessment.

For patients with chronic conditions like CHD, it is evident that further multi-centre studies, involving larger patient cohorts and longer follow-up, are required to better understand the true estimate of CED accrued across their lifetime. Implementation of dose reduction measures should be applied more rigorously by those involved in their care to reduce unnecessary radiation and ultimately reduce their chance of developing a radiation-induced cancer.

## 5. Conclusions

It has long been accepted that children are at a higher risk than adults for radiation-induced cancer. This review has demonstrated that the radiation burden from medical imaging is elevated in paediatric patients with congenital heart disease, with radiation from diagnostic and interventional cardiac catheterisation representing the greatest cumulative dose. Overall, the importance of following ALARA principles is vital. Efforts on dose optimisation, limiting radiation exposure, and the use of alternative modalities without ionising radiation where possible are crucial to minimise lifetime CED in this cohort.

## Figures and Tables

**Table 1:** Summary of included studies and cumulative effective radiation.

Author (Year)	Study Type	Number of Participants	Age of Participants, Years	Radiation Source	Overall Cumulative Effective Dose (mSv)	^1^ Participants Cumulative Effective Dose >20 mSv
Ait-Ali (2010) [22]	Prospective cohort	59	2.8 (mean)	All ionising radiation	7.7 (median)	0/59
Jones (2017) [23]	Retrospective cohort	117	0–17 (range)	Interventional procedures	16.5 (median)	14/117
Ubeda (2019) [24]	Prospective/ retrospective cohort	1521	2–8 (range)	Interventional procedures	8.7 (>four procedures) (mean)	0/1521
McDonnell (2014) [25]	Retrospective cohort	31	13.6 (median at heart transplant)	All ionising radiation	53.5 (mean)	Unknown
Glatz (2014) [26]	Retrospective cohort	4132	0.3 (mean)	All ionising radiation	0.96 (median)	218/4132
Downing (2015) [27]	Retrospective cohort	38	^2^ Birth-Fontan closure	All ionising radiation	25.7 (mean)	2.9
Johnson (2014) [28]	Retrospective cohort	337	0.24 (median at heart transplant)	All ionising radiation	2.67 (median)	94/337

^1^ 20 mSv used as it is the current limit on effective dose for occupational exposure. ^2^ Fontan completion is a procedure to re-route the systemic deoxygenated blood from the venous circulation into the pulmonary vasculature.

## Data Availability

Not applicable.

## Supplementary Materials

The following supporting information can be downloaded at: https://www.mdpi.com/article/10.3390/children10040645/s1, Table S1: Excluded studies after full-text review [38,39,40,41,42,43,44,45,46,47,48,49,50,51]; Table S2: Contribution of total radiation exposure from various imaging modalities.

## Abbreviations

ALARA—As low as reasonably achievable; CAHD—congenital and acquired heart disease; CED—cumulative effective dose; CHD—congenital heart disease; CT—computed tomogram; DRL—diagnostic reference level.
