# Supplementary material for: Cumulative Radiation Dose from Medical Imaging in Children with Congenital Heart Disease: A Systematic Review
Source: Children (Basel). 2023 Mar 30;10(4):645. doi: 10.3390/children10040645 (PMC10136955; doi:10.3390/children10040645)
Supplement: Supplementary file 1 [file children-10-00645-s001.zip › children-2264902-Supplementary.pdf]

Table S1: Excluded studies with reasons following full-text review.

Author (year)	Summary comment for exclusion
El Sayed (2012) [39]	No time scale for collected data
Glatz (2014) [40]	Includes patients >18 years old
Kawasaki (2015) [41]	Phantoms utilized
Keiller (2015) [42]	No cumulative data
Manica (2020) [43]	No cumulative data
Kottou (2018) [44]	No cumulative data
Harbron (2015) [45]	Includes patients >18 years old
Martinez (2007) [46]	Outside search limit timeline
Paul (2011) [47]	<1 year data
Walsh (2015) [48]	No cumulative data
Watson (2013) [49]	<1 year data
Yakoumakis (2013) [50]	No cumulative data
Kobayashi (2014) [51]	No cumulative data

Table S2. Contribution of total radiation exposure from various imaging modalities.

Author (year)	Contributions of various procedures
Ait-Ali (2010) [22]	X-rays represented >95% of examination and contributed 5% of cumulative dose<br>CT represented 1% of examination and contributed 11% of cumulative dose<br>Diagnostic catheterisations represented 3.5% of examinations and contributed 41% of cumulative dose<br>Interventional catheterisations represented 2.5% of examination and contributed 43% of cumulative dose
Jones (2017) [23]	Interventional cardiac procedures only
Ubeda (2019) [24]	Cardiac procedures only
McDonnell (2014) [25]	28 X-ray examinations on average per patient contributed 3% of cumulative effective dose (1.9 mSv)<br>Eight catheterisations on average per patient contributed 91% of cumulative effective dose (47.5 mSv)
Glatz (2014) [26]	Cardiac catheterizations represented a small fraction of overall examination count, but a large share of the cumulative radiation exposure came from these procedures
Downing (2015) [27]	Two catheterizations on average per patient contributed 78% of annual effective dose (6.3 mSv/yr)<br>Two IR and fluoroscopy examinations on average per patient contributed 1% of annual effective dose<br>53 X-ray examinations on average per patient contributed 8% of annual effective dose (0.6 mSv/yr)
Johnson (2014) [28]	Catheterizations represented <5% of examinations and contributed 60% of cumulative dose<br>CT represented <5% of examinations and contributed 20% of cumulative dose<br>Fluoroscopy represented <5% of examinations and contributed <10% of cumulative dose<br>Nuclear Med represented 1% of examinations and contributed <5% of cumulative dose

References:

38. El Sayed MH, Roushdy AM, El Farghaly H, El Sherbini A. Radiation exposure in children during the current era of pediatric cardiac intervention. Pediatr Cardiol [Internet]. 2012;33[1]:27–35.
39. Glatz, A.C.; Patel, A.; Zhu, X.; Dori, Y.; Hanna, B.D.; Gillespie, M.J.; Rome, J.J. Patient Radiation Exposure in a Modern, Large-Volume, Pediatric Cardiac Catheterization Laboratory. *Pediatr. Cardiol*. **2014**, *35*, 870–878.
40. Kawasaki T, Fujii K, Akahane K. Estimation of Organ and Effective Doses for Neonate and Infant Diagnostic Cardiac Catheterizations. Am J Roentgenol [Internet]. 2015 Dec 10;205[3]:599–603.
41. Verghese GR, McElhinney DB, Strauss KJ, Bergersen L. Characterization of radiation exposure and effect of a radiation monitoring policy in a large volume pediatric cardiac catheterization lab. Catheter Cardiovasc Interv. 2011/04/28. 2012;79[2]:294–301.
42. Keiller DA, Martin CJ. Radiation dose to the heart in paediatric interventional cardiology. J Radiol Prot [Internet]. 2015;35[2]:257–64
43. Manica JL, Duarte VO, Ribeiro M, Hartley A, Petraco R, Pedra C, et al. Standardizing Radiation Exposure during Cardiac Catheterization in Children with Congenital Heart Disease: Data from a Multicenter Brazilian Registry. Arq Bras Cardiol. 2020;115[6]:115.
44. Kottou S, Kollaros N, Plemmenos C, Mastorakou I, Apostolopoulou SC, Tsapaki V. Towards the definition of Institutional diagnostic reference levels in paediatric interventional cardiology procedures in Greece. Phys Medica [Internet]. 2018;46:52–8.
45. Harbron RW, Pearce MS, Salotti JA, McHugh K, McLaren C, Abernethy L, et al. Radiation doses from fluoroscopically guided cardiac catheterization procedures in children and young adults in the United Kingdom: a multicentre study. Br J Radiol. 2015;88[1048]:20140852.
46. Martinez LC, Vano E Fau - Gutierrez F, Gutierrez F Fau - Rodriguez C, Rodriguez C Fau - Gilarranz R, Gilarranz R Fau - Manzanas MJ, Manzanas MJ. Patient doses from fluoroscopically guided cardiac procedures in pediatrics. Phys Med Biol. 2007;52[16]:474.
47. Paul J-F, Rohnean A, Elfassy E, Sigal-Cinqualbre A. Radiation dose for thoracic and coronary step-and-shoot CT using a 128-slice dual-source machine in infants and small children with congenital heart disease. Pediatr Radiol. 2011;41[2]:244–9.
48. Walsh MA, Noga M, Rutledge J. Cumulative radiation exposure in pediatric patients with congenital heart disease. Pediatr Cardiol. 2015;36[2]:289–94.
49. Watson TG, Mah E, Joseph Schoepf U, King L, Huda W, Hlavacek AM. Effective Radiation Dose in Computed Tomographic Angiography of the Chest and Diagnostic Cardiac Catheterization in Pediatric Patients. Pediatr Cardiol [Internet]. 2013;34[3]:518–24.:
50. Yakoumakis E, Kostopoulou H Fau - Makri T, Makri T Fau - Dimitriadis A, Dimitriadis A Fau - Georgiou E, Georgiou E Fau - Tsalafoutas I, Tsalafoutas I. Estimation of radiation dose and risk to children undergoing cardiac catheterization for the treatment of a congenital heart disease using Monte Carlo simulations. Pediatr Radiol. 2013;43[3]:339-.
51. Kobayashi D, Meadows J, Forbes TJ, Moore P, Javois AJ, Pedra CA, et al. Standardizing radiation dose reporting in the pediatric cardiac catheterization laboratory—a multicenter study by the CCISC [Congenital Cardiovascular Interventional Study Consortium]. Catheter Cardiovasc Interv. 2014;84[5]:785–93.
